# Service encounters and the manufacture of harm: violence in the service economy under late-modernity

**DOI:** 10.3389/fsoc.2026.1782224

**Published:** 2026-03-25

**Authors:** Liam Miles, Mikahil Azad

**Affiliations:** 1 Department of Criminology, University of Northampton, Northampton, United Kingdom; 2 Department of Criminology, University of Worcester, Worcester, United Kingdom

**Keywords:** harm, late modernity, ontological insecurity, risk society, service economy, violence

## Abstract

This article examines the proliferation of violence within Britain’s service economy, focusing on delivery drivers. The discussion and analysis will examine three recent acts of violence against delivery drivers and framed as part of a wider continuum of violence against service economy workers. Although the presented cases occurred as direct acts of violence, it is the responses of the delivery drivers who faced these attacks that is centre ground to our inquiry. We argue the responses of the delivery drivers, to safeguard their courier goods is symptomatic of a risk society, discussed by Ulrich Beck fuelled by Anthony Gidden’s idea of perpetual ontological insecurity. The conceptual marriage here between Beck and Giddens enables us to present the conditions of the service economy. As labour market security becomes uncertain, workers experience hyper-competition and precarity that ostensibly produces an insecurity, thus provoking these workers to engage in risk-taking behaviours to maintain economic and social security. This article concludes that violence in the service economy may be enabled by structural processes and has become culturally legitimated, rather than exceptional.

## 1 Introduction

This article adopts a multiple case study approach (Yin, 2014) to explore the phenomenon of violence against delivery drivers. These workers form part of the wider service economy, including retail staff, night-time economy workers, and fast-food workers. According to Robinson (2025), there were approximately 1,195 reports of violence committed against service-economy workers collectively in 2024. Fundamentally, this represents only the cases reported and recorded by the British Safety Council and is unreflective of the true scale of this issue. Moreover, as will be unpacked in the literature review, the phenomenon of violence against service-economy workers has begun to receive significant scholarly attention. In this article, we recognise the valuable contribution of empirical studies toward advancing structural explanations for violent crimes against service-economy workers (Winlow, 2001; Lloyd, 2018; Lynes and Wragg, 2023; Bushell and Braithwaite, 2024). This article also recognises the earlier scholarly contributions to understanding violence beyond the interpersonal, as constitutive of structural processes (Galtung, 1969), that can become embedded in culture (Galtung, 1990) and normalised as part of reality (Zizek, 2009). These ideas offer a suitable framework for harnessing Beck’s (1992) idea of risk society and ontological insecurity (Giddens, 1991), which we consider for theorising the phenomenon of violence against service-economy workers.

### 1.1 Late-modernity—the rise of the service economy and manufactured risk

This section brings together vital commentary on how British society has changed since the neoliberal turn of the 1970s. These changes include individualisation (Young, 1999), flexibilisation of labour markets (Harvey, 2005), and a rising service economy, as consumer culture rose exponentially (Hayward, 2004). These changes are situated by Giddens (1991) and Beck (1992) as the coming of Late-Modernity. This article adopts Late-Modernity as a conceptual framework to examine how the phenomenon of violence against service-economy workers can be addressed.

Following the neoliberal turn of the mid-1970s, Britain underwent a seismic process of social and economic restructuring that led to the rapid decline of many industries, including manufacturing (Martin, 1986) and steelmaking (Tomlinson, 2021), during the first wave of the Industrial Revolution (Marren, 2016). Globalisation accelerated exponentially during the 1990s leading to the cheaper labour procurement, often sourced from outside Britain (Gregory, 2002). During this time, Britain, like America, began to import goods and services more than to export. According to Black (2004), this contributed to the rise of a consumer culture in Britain.

Numerous commentators (Giddens, 1991; Beck, 1992; Young, 1999, 2007; Bauman, 2000; France, 2007) argue that under conditions of globalised free-market capitalism, Western society has entered a stage of Late-Modernity. The acceleration of globalisation and the birth of the neoliberal state are significant processes of social, economic, and political restructuring that have shaped the boundaries and conditions of capitalism under Late-Modernity (Bauman, 1998; Beck, 1992). Through this, Beck et al. (1994) argued society has entered a stage of reflexive modernisation. As labour markets became ever more insecure, so too did the life trajectories of many people, thus provoking a reflexivity as to how one might secure their future. The precarious and hyper-competitive conditions of the service economy have replaced secure labour market functionalities, thus producing new risks.

The reflexive turn can be argued to be a part of a wider process of individualisation and precarity Beck and Beck-Gernsheim (2002), as labour markets entered a stage of flexibilisation. Following the collapse of Keynesian welfare economics in the late 1970s, neoliberalism occupied a stronghold within Britain’s basis for political economy, with an ever-closer agenda toward globalism (Martell, 2008). Historically, the world has always possessed a global outlook, particularly in relation to nation-state relations, trade, and migration (Krueger, 2003; Amadi, 2020). Significantly, however, it is the acceleration of globalised socio-economic restructuring processes that constitutes globalisation as it is known today (Bauman, 1998). For Voisey and O’Riordan (2001) time and space have converged, enabled by a democratisation of air travel, trade, and the liberalised flow of goods, services, and people.

Beck (1992) suggested that globalisation and the liberalisation of the market economy have brought forth a risk society. The nature of these risks can be attributed, but not limited to, the increased threat of terrorism, disease, pandemics, and poverty enabled by global flows of movement. Risk, for Beck (1992), is also the collapse of the welfare state and insecure labour markets. Although Beck (1992) defined what may constitute risk, this article suggests that the nature of risks can change over time, reflecting the contemporary condition. The service economy, for example, has produced a new layer of risk for workers. This will be critically addressed later, when empirical case studies are foregrounded. Moreover, these new layers of risks will be considered as manifestations of ontological insecurity (Giddens, 1991).

It is important to note that the term ‘ontological insecurity’ was originally applied by Laing (1960), who observed healthy patients diagnosed with Schizophrenia. For Laing (1960: 68), to be ontologically secure is to ‘encounter, embrace and respond to risks life poses’. Significantly for Laing, this includes boundaries within a social, ethical, spiritual, and biological nature. Later, Giddens (1991, 1994) and Bauman (1998) departed from Laing (1960) idea of ontological insecurity as a pathological condition and turned to political economy. Giddens (1991) observed the rise of consumer capitalism, proliferated by globalisation and the liberalisation of the market economy, which has placed people into social and economic insecurity. Situating ontological insecurity as a ‘process of being’ (Giddens, 1984), capitalism under Late-Modernity has reoriented an understanding of what ontological insecurity may be. Giddens questioned how the duality between structure and agency has enabled an ontological insecurity amongst people. Therefore, to be ontologically insecure can mean being at a loss for control over one’s agency in response to structures (consumer culture) that shape agential powers. According to Giddens (1991), facets of insecurity can be found in the breakdown of routine, particularly in modes of employment. When workers lose a sense of job security, ontological insecurity may be present and could shape decision-making. Meanwhile, Bauman (1998) questioned the meaning behind ontological insecurity. Drawing upon Kierkegaard’s existentialist idea of ‘angst’, Bauman proposed that to be ontologically insecure is to hold a perpetual anxiety over one’s sense of self. The backdrop of these conditions is important for understanding conceptualisations of violence.

Kinnvall (2017), who wrote from a post-colonial discourse, challenged Giddens’s framing of ontological insecurity and suggested that the construction of insecurities produces a populist discourse, particularly on matters involving migration, state sovereignty, and social cohesion. Ontological insecurity may therefore be considered as a political imaginary. Irrespective of this, however, the risks and insecurities found in Britain under Late-Modernity can be observed. This involves a fall of secure labour markets Beck et al. (1994), a breakdown of social and community relations (Bauman, 2000), and a polarisation of citizens from the political space (Winlow and Hall, 2023). Therefore, it can be considered myopic to ignore or dilute the significance of people’s lived realities in association with their social security and belonging. In summary, this section has closely examined the idea of Late-Modernity, risk, and insecurity. These contextual layers will help shape a critical understanding of violence and the service economy.

### 1.2 Violence and the service economy

This section addresses structural and cultural explanations of violence (Galtung, 1969, 1990) that transcend the interpersonal. This section will move with these ideas to understand violence in the service economy. Violence is multi-layered, complex, and an idea that has received significant academic attention. This has fundamentally emerged from two schools of thought. The first is understanding physical acts of violence within biological explanations (Denno, 2007; Raine, 2013; Rollins, 2021; Siann, 2024). Here, violence is understood as a physical encounter, reproductive of a pathological and biological condition.

The second approach is structural (Short, 1997; Hall, 1997; Savolainen, 2010; Lymperopoulou and Bannister, 2022). There is significant commentary suggesting violence can be precipitated by structural conditions such as labour market restructuring and poverty. Here, physical violence can be considered as an instrument for the individual to ascertain power, in a time of seeming powerlessness- be that social ostracisation, economic marginalisation, and political disempowerment (Hall, 1997). The term ‘structural violence’ was then coined by Galtung (1969) to identify acts of violence that exist in everyday life. Much of this understanding situates violence as a physical or direct act being committed by an unknown actor, as consequential to the conditions they are placed under. Galtung (1990) later developed the idea of cultural violence. Galtung (1990:1) defined cultural violence as ‘any aspect of a culture that can be used to legitimise violence in its direct or structural form’. From this, cultural violence can be seen to further sustain these systemic issues by providing ideological justifications for both direct and structural violence. Galtung (1990) emphasised that cultural violence is embedded in language, religion, media, and education, thus shaping perceptions that normalise acts of harm and inequality. Galtung (1990) refers to the concept of a ‘master-race’, such as what was seen throughout the Nazi Holocaust during the Second World War, that can be posited as an act of cultural violence.

Through this understanding, violence may not be considered as an occurrence in isolation from the event itself, but rather as an expression of ideology sustained by societal structures. Understanding these interconnections is crucial to developing ideas beyond reactive measures to addressing everyday acts of violence in society. Zizek (2009) supports this idea and suggests that understanding direct violence without recognition of its structural and cultural foundations can lead to superficial reasoning that fails to disrupt deeper cycles of systematic oppression. This comprehensive perspective underscores the necessity of addressing the root causes of violence rather than merely its symptoms.

This section will now turn to violence within the UK service economy. There is a growing presence of academic literature focused on violence in the service economy, and substantially, this attention is filtered down into three key areas. The first is the service economy broadly (Bishop et al., 2005; Korczynski and Evans, 2013; Lloyd, 2018). The second is violence and harm against retail staff (Rayner et al., 2002; Taylor, 2022; Bushell and Braithwaite, 2024). The third is violence in the Night-Time Economy (NTE) (Winlow, 2001; Hobbs et al., 2005; Lister et al., 2010). On violence against couriers and warehouse workers, including at companies such as Amazon, there has been limited scholarly focus. However, Lynes and Wragg (2023) have offered substantial commentary in relation to structural modes of violence committed against Amazon warehouse workers, under the gaze of capitalism in Late-Modernity.

According to Taylor (2022), violence against retail workers across the UK has risen exponentially. Taylor (2022) situates the coronavirus disease 2019 (COVID-19) pandemic as a key precipitator behind the rise of violent crime in the retail sector, particularly at a time of societal panic fuelled by restrictions on people’s movement. Meanwhile, Bushell and Braithwaite (2024) recognise the role of the pandemic and suggest that there are additional explanations for this rising tide of violence beyond the pandemic—namely, this is consumer culture.

The emergence of a consumer culture marked a new age of accelerated individualism (Young, 1999). The substance of daily life rapidly evolved into the democratisation of goods and services, of which the consumer faced an abundance of choice. This differed from the values of consumption in the post-war period, which was formally led by the need to ration, ‘live within one’s means, and express gradual gratification (Fenwick and Hayward, 2000). Meanwhile, the values expressed under a consumer culture are underpinned by immediate gratification and hedonistic excess (Raymen and Smith, 2016). Ostensibly, this has led to social change, as people increasingly pursue their desires, knowing that their actions may harm others. Hall (2012) refers to this as Special Liberty and has been used to address the proliferation of harm in the service economy (Lloyd, 2018; Bushell and Braithwaite, 2024). Similarly, Billingham and Irwin-Rogers (2022) discuss the idea of ‘mattering’, the idea that people may be considered disposable based on their social, economic, and symbolic status. Often, this is led by a person’s occupational status and may be used to explain why some people in society face a disproportionate likelihood of encountering violence.

### 1.3 Conclusion

This section has addressed three important ideas. The first is how society in Britain has entered the stage of Late-Modernity (Giddens, 1991). This is characterised by the acceleration of globalisation, neoliberalism, and consumer capitalism, leading toward the dominance of the service economy and the flexibilisation of labour, which, according to Beck (1992), has led to a risk society, provoking individuals to exercise reflexivity regarding their life trajectories. Giddens (1991) frames these moments of social and economic restructuring as leading to ontological insecurity. The backdrop of these conditions is what this article foregrounds as underpinning moments of violence.

Section 2.2 explored Galtung (1969) idea of structural violence and his later work on cultural violence in 1990. This can be understood as ideologically manifested (Zizek, 2009). The presented case studies will provide an empirical basis for situating violence as an encounter beyond the physical. Finally, this section explored the proliferation of violence in the service economy and how such a theoretical integration can be harnessed to make sense of violence against service-economy workers as a rising phenomenon. The following section will outline the chosen methods to further situate and justify the research approach.

## 2 Methodology

The purpose of this article is to offer an analysis of violence against service-economy workers. Late-Modernity has been used as a conceptual framework for understanding violence. This framework utilises Beck’s (1992) idea of risk society and Giddens (1991) basis of ontological insecurity. This conceptual framework will be supported by empirical case studies to foreground real, contemporary incidents of violence, often underpinned by acquisitive crime, as a basis for evidence. The chosen method is a multiple case study approach (Yin, 2014) of news articles in Britain detailing incidents of violence against service-economy workers.

Yin (2009: 18) defines a case study approach as.

*‘An empirical inquiry which investigates a phenomenon in its real-life context’.*

Similarly, (Creswell, 2014, 241) situates case study approaches as a.

*… ‘qualitative design in which the researcher explores in depth a program, event, activity, process or one or more individual’.*

Although numerous articles detailing violence against service-economy workers were discovered (see Supplementary Appendix A), three news articles have been selected to situate the analysis. This is for three reasons. First, it is not our intention to analyse the discourse behind the media reporting of these incidents. It is the objective facts of these incidents, including the nature of the attack, timing, location, and method, that are explored. Second, a multiple case study approach is considerably more rigorous than a single case study approach, as evidence can be weighed against one another, thereby removing the idea that a single case in isolation is merely an anomaly (Priya, 2020). This idea is supported by Yin (2014), who explores the media as a suitable example of such case study analysis.

The limitations of the media, however, are recognised, particularly in fuelling moral panics for profiteering (Horsley, 2017; Kelly et al., 2020). The use of these articles has not formed as part of the analysis, only as a description of the events that occurred in accordance with the media sources and the reported legal proceedings. Therefore, it is timely to emphasise that this article is not conducting a discourse analysis on how the media reports the crime. Alternatively, the media reports are applied as case study examples demonstrating cases of violence against service-economy workers.

The procedural method involved searching numerous news databases, including BBC News and Sky News, and Local News channels, including Birmingham Mail, to identify and locate case studies involving acts of violence committed against service-economy workers. Through a coding process, the key observations and findings from the articles were put into a document and clustered. These themes built the basis for the following discussion sections. The following section will unpack the three case studies, offer an account of what occurred, and then will be built into a wider body of analysis.

## 3 An overview of the case studies

The following cases detail contemporary moments involving acts of violence and harm against service-economy workers.

### 3.1 Case study 1

According to Banks (2024), two brothers—Daniel Conray, aged 39, and Sean Conray, aged 37—launched an opportunistic attack on an Asda delivery driver, after attempting to steal a crate of lagers, as the victim (unnamed) was unloading crates from the delivery van. Upon noticing the attempted theft of the lagers, the victim confronted Daniel and Sean and attempted to stop their theft. In response, Daniel and Sean attacked the delivery driver, leading him to sustain a ‘split lip, bruising and swelling to his face, lacerations to his knee and a black eye’ (Banks, 2024). At this time, Daniel and Sean rode away on their bikes, with the stolen lager. It was also reported that the victim of this attack experienced heightened levels of anxiety upon returning to work.

### 3.2 Case study 2

According to a report by Sky News (2025), an Amazon delivery driver called Mr. Konder was killed after sustaining injuries to his chest and head. This was following the robbery of his van committed by Mr. Mark Ross. On 20 August 2024, Mr. Konder was making deliveries in Leeds. At this time, Mr. Ross, who, according to Sky News (2025), was on his way to buy cannabis, saw the van unattended, entered the vehicle, and drove off. The intention of this act remains unknown. Mr. Konder clung onto the back of the van, which dragged him for several minutes across the street before being crashed by Mr. Ross into two parked cars, leading to the death of Mr. Konder.

### 3.3 Case study 3

In May 2025, an unnamed delivery driver in Kent was attacked by Mr. Shaun Mitchell, who, according to Langridge (2025) report, ‘grabbed her clothing and dragged her out of the van before throwing her to the floor’. Also, according to this report, ‘The 48- year-old then jumped into the vehicle and attempted to turn on the ignition. When the engine did not start, the victim reached in and pulled out the keys before fleeing to a nearby house. Mitchell then stole some parcels from the passenger seat and left on foot*’.*

## 4 An integrated discussion

These three cases seemed to occur in the pursuit of acquisitive gain. The means to achieve this resulted in direct violence against the delivery driver. Case study 1 involved a violent attack to acquire a crate of lagers. Similarly, in case study 3, Mr. Mitchell attacked a delivery driver to steal parcels. These incidents all demonstrate acts of physical violence. It can be theorised that offenders’ actions are symptomatic of a consumer culture (Hayward, 2004) that permits people to actualise their desire for a moment of satisfaction and fulfilment. Equally, Hall (2012) idea of Special Liberty is significant to understand how, under consumerism, harm may be inflicted on others, for the fulfilment of one’s own desire. This signifies a void or ontological insecurity (Giddens, 1991) that is addressed through violence. However, the delivery driver’s intervention to prevent the goods from being stolen can be understood as symptomatic of life in a risk society (Beck, 1992). The question, however, is what these risks consist of.

As discussed by Lloyd (2019), service-economy workers face significant risks to their physical safety. Additionally, these workers face risks to their livelihood, particularly when targets are not met. This is supported by Lynes and Wragg (2023) study on Amazon warehouse drivers, which found that workers were exposed to meeting targets with punitive sanctions if they failed to do so. In the context of case study 1, the victim was a courier for Asda. According to the Asda website [see Asda (2025)], delivery drivers are often placed onto permanent contracts, ensuring a heightened level of job security, as compared to those working for couriers such as Uber Eats and Deliveroo. However, following an examination of various reviews from Asda employees for delivery drivers (Indeed.com, 2025), numerous results came back negatively. Feedback detailed poor management, a lack of worker autonomy, and an increased pressure on delivery drivers to meet their daily targets, irrespective of external factors, including workers’ health, family commitments, and traffic delays. If targets are not met, as a statutory requirement for the job (See the Asda website), the worker may be subject to performance reviews, which, as an outcome, could see the worker’s contract being terminated.

The liberalisation of work has led to the rise of platform-based and gig-economy work. This can be observed in the rise of companies such as Amazon, DPD, Deliveroo, Uber, and Bolt. For Lloyd (2018), these labour markets operate to prioritise cost-efficiency and customer satisfaction over the safety and dignity of workers. This is demonstrated by the emergence of a firing-and-hiring culture. Beck (1992) noted this shift and suggested this has produced significant risks for workers. Beck et al. (1994) furthered this, attributing the casualisation of labour to driving people into a state of reflexivity in order to securing their future.

Meanwhile, case study 2 involving the death of Mr. Konder signifies similar motivations and consequences attuned to life in a risk society. This is reflected in the juxtaposition between his response to the robbery, which ultimately escalated matters, and Amazon’s written policy for protecting workers. According to the Amazon website (See Amazon Jobs.com), all workers are afforded safe working conditions, including the right to refuse service delivery if the conditions feel unsafe. It is important, however, to understand that Amazon is a leading example of contemporary labour market liberalisation (Lynes and Wragg, 2023) as its workers can face risk and insecurity. Namely, this can be observed through low wages, unsafe working conditions, constant algorithmic monitoring of performance, enabled by the Artificial Intelligence revolution (Lynes and Wragg, 2023). Amazon workers are subjected to punitive measures designed to maximise productivity and regulate their behaviour, including bathroom breaks, to ensure maximum productivity. Here, it can be noted that the engagement in risk-taking behaviours may be observed by some Amazon drivers, including speeding on the roads and parking in impractical and dangerous spaces, with little opportunity for rest (Tims, 2019).

In case study 2, Sky News (2025) reported that Mr. Konder ran and clung onto the back of the van in what seemed to be an attempt to stop Mr. Ross stealing the goods. Although the true intentions of Mr. Konder will never be fully known, it can be suggested that the fear of sanction if the goods were lost may have fuelled Mr. Konder’s decision to go above and beyond to confront Mr. Ross. Therefore, the risk of being fired due to failure to meet targets and safeguard goods is symptomatic of Beck’s (1992) risk society, as failing to perform and hit targets may result in job loss and economic insecurity (Giddens, 1991). This arguably may offer a rationalisation behind the decision-making of the delivery drivers presented in these three case studies.

Mr. Konder’s actions were described by the sentencing judge as an act that positioned him as ‘Expendable’ (Sky News, 2025). It can be inferred that to label someone as ‘expendable’ means to hold that person in little significance and can therefore be removed. The notion of disposability amongst service-economy workers has received limited scholarly attention (Bales, 1999; Rodkey, 2016; Henaway, 2023), however, it can be maintained that when harm occurs that is directed at service-economy workers, these notions of disposability ought to be considered.

Billingham and Irwin-Rogers (2022) idea of ‘mattering’ can be applied to understand this phenomenon of violence. Mattering is considered a relational concept, underpinned by existential questioning toward how an individual places their sense of worth into society. Matters of occupation, income level, class, and ethnicity can shape a picture of one’s worth in society. Perhaps this might explain why, upon meeting a new person, the first question we tend to ask is ‘what do you do’? The concept of ‘mattering’ (Billingham and Irwin-Rogers, 2022) may be harnessed to understand this instinctual process of rapid evaluation to determine if an individual matters and is worthy of our time, attention, and respect. It is suggested that delivery drivers face an increased risk of violence, because the perpetrator may not consider them worthy of mattering. However, it is argued that the underbelly of neoliberal capitalism, in shaping working conditions and some public attitudes toward service-economy workers, has produced a dichotomy between consumer and producer values. The desires of the consumers can be met through engaging in a consumer process that responds to and satisfies the wish for instant gratification, hedonistic excess, and insatiability of desire (Hayward and Smith, 2017). Meanwhile, for the worker, as argued by Lloyd (2018), there is significant pressure to ensure all the consumer demands are met. Moreover, for Lloyd (2018), companies such as Amazon seek to ensure on-time deliveries. This target-and-measure approach signifies a risk (Beck, 1992) that the worker may encounter if they do not meet targets. Living under a risk society can come with cultural violence (Galtung, 1990), embedded into the working and living conditions of daily life. For workers in the service economy, this may hold significant implications. This may include the normalisation of insecurity, the glorification of hustle culture, and the minimisation of labour rights, contributing to an environment where workers’ lives are not fully valued or protected.

## 5 Conclusion

This article has adopted three recent case studies of violence against delivery drivers in the UK as part of a wider service economy. It has been argued that the actions taken by the delivery drivers to protect their goods from being stolen are symptomatic of a risk society (Beck, 1992). Fear of losing one’s job is part of living in a risk society and can drive decision-making. This is upheld by punitive working conditions, such as monitoring progress and docking pay if targets are not met (Lynes and Wragg, 2023). Moreover, risk can be understood in relation to neoliberal labour market restructuring, leading to insecurity (Lloyd, 2018). These three case studies suggest that, in capitalism under Late-Modernity, violence can be understood as part of a system, embedded in structural (Galtung, 1969) and cultural (Galtung, 1990) conditions. Therefore, these cases ought not to be considered anomalies but as a testament to the rising condition of ontological insecurity (Giddens, 1991) propagated under a risk society (Beck, 1992). These events should prompt a critical reflection on the working conditions in the service economy and demand urgent policy and social responses. As digital economies expand, future research must investigate how algorithmic management, surveillance technologies, and customer-rating systems contribute to new forms of harm and control. Equally, attention must be paid to how gig workers mobilise resistance, build solidarity, and challenge the logics of disposability imposed upon them. Without such analysis, violence against workers will remain an ongoing yet underacknowledged symptom of our current economic order.
