# Supplementary material for: Service encounters and the manufacture of harm: violence in the service economy under late-modernity
Source: Front Sociol. 2026 Mar 25;11:1782224. doi: 10.3389/fsoc.2026.1782224 (PMC13056631; doi:10.3389/fsoc.2026.1782224)
Supplement: Supplementary file 1 [file Supplementary_file_1.pdf]

Appendix A

Author	Date	News Article Source	News Article Title	URL Link	Summary
Paul Jeeves	19th November 2019	Daily Express	Delivery drivers face huge rise in abuse and theft in takeaway chaos	https://www.express.co.uk/news/uk/1977922/delivery-riders-face-increasing-abuse	Demands by delivery drivers to have more protection by their employers and by the state, in response to rising rates of violence
Emily Cooper<br><br>Hannah Boland	20th August 2024<br><br>16th August 2024	Daily Mail<br><br>The Telegraph	Tesco arms delivery drivers with DNA 'Spit Kits' to help trace abusive customers amid rise in violence	https://www.dailymail.co.uk/news/article-13760559/Tesco-delivery-drivers-DNA-kits-help-abusive-customers-violence.html<br><br>https://www.telegraph.co.uk/business/2024/08/16/tesco-gives-delivery-drivers-dna-tests-abusive-customers/#:~:tex	Tesco have provided delivery drivers DNA 'Spit Kits' following a rise of attacks against delivery drivers.

				t=Britain's%20biggest%20supermarket%20is%20understood,aggressive%20and%20spit%20at%20them<br><br>https://www.telegraph.co.uk/business/2024/08/16/tesco-gives-delivery-drivers-dna-tests-abusive-customers/#:~:text=Britain's%20biggest%20supermarket%20is%20understood,aggressive%20and%20spit%20at%20them.	
Polly Smythe	7th August 2024	Novara News	Uber and Deliveroo Drivers and fearing for their lives and the apps couldn't care less.	https://novaramedia.com/2024/08/07/uber-and-deliveroo-drivers-are-fearing-	Far right violence has forced delivery drivers to choose between income and safety.

				for-their-lives-and-the-apps-couldnt-care-less/	
Ben Haslam	13th November 2024	Liverpool Echo	Man 'didn't leave house for a week' following violent, racist abuse.	https://www.liverpoolecho.co.uk/news/liverpool-news/man-didnt-leave-house-week-30326941	A delivery driver 'Afnan Khan' was racially abused after just doing his job.
Eloise Hill	25th November 2024	Retail Gazette	BRC urges public to stand against retail worker abuse this Christmas	https://www.retailgazette.co.uk/blog/2024/11/brc-safe-christmas/	The British Retail Consortium (RBC) urges the public to stand in solidarity with retail workers following a rise in violent incidents and abuse.
Georgia Banks	25th September 2024	Teesside News	Boozed up Thornabny brother violent robbery on Asda delivery worker	https://www.gazettelive.co.uk/news/teesside-news/boozed-up-thornaby-brothers-violent-29996220	Two brothers, drunk on alcohol attacked an Asda delivery driver and stole a crate of lagers.
Sarah Marsh	11th June 2024	The Guardian	One in three Asda staff have been attacked at work, survey finds	https://www.theguardian.com/business/article/2024/jun/11/asda-staff-	A survey from the Guardian revealed the rate in which Asda workers have faced violence at work, including delivery drivers.

				attacked-at-work-gmb-poll	
